# 

**Published:** 1882-05

**Authors:** 


Eye and Ear Infirmary—1.15 p. m., Otological, by Prof. Jones; 2.15 p. m., Ophthalmological, by Prof. Hotz.

Mercy Hospital—2 p. m., Medical, Profs. Hollister and Quine.

Rush Medical College—3 p. m., Dermatological and Venereal, by Prof. Hyde.

Woman's Medical College—2 p. m., Dermatological and Venereal, by Prof. Maynard.

TUESDAY.

Cook Co. Hospital—2 to 4 p. m., Medical and Surgical Clinics.

Mercy Hospital—2 p. m., Surgical Clinic, by Prof. Andrews.

Woman's Medical College—10 a. m., Prof. Ingals.

WEDNESDAY.

Chicago Medical College—2 p. m., Eye and Ear, by Prof. Jones.

Rush Medical College—2 p. m., Medical, by Dr. Bridge; 3 p. m., Ophthalmological and Otological, by Prof. Holmes; 3:30 to 4:30 p. m., Diseases of the Chest, by Dr. E. Fletcher Ingals.

Woman's Medical College—2 p. m., Eye and Ear, by Dr. W. T. Montgomery.

Eye and Ear Infirmary—2.30 p. m., Dr. E. J. Gardiner.

THURSDAY.

Chicago Medical College—2 p.m., Gynæcological, Prof. Dudley.

Rush Medical College—2 p. m., Diseases of Children, by Dr. Knox; 3 p. m., Diseases of the Nervous System, by Prof. Lyman.

Eye and Ear Infirmary—2 p. m., Ophthalmological, by Dr. Hotz.

Woman's Medical College—3 p. m., Surgical, by Prof. Owens.

FRIDAY.

Cook County Hospital—2 to 4 p. m., Medical and Surgical Clinics, by Profs. J. H. Hollister and R. N. Isham.

Mercy Hospital—2 p. m., Medical, by Prof. Davis.

SATURDAY.

Rush Medical College—2 p. m., Surgical, by Prof. Gunn.

Mercy Hospital—2 p. m., Surgical Clinic, by Prof. Andrews.

Chicago Medical College—3 p. m., Neurological, Prof. Jewell.

Woman's Medical College—2 p. m., Gynæcological, by Prof. Stevenson.

Daily Clinics, from 2 to 4 p. m., at the Central Free Dispensary, and at the South Side Dispensary.

